# Supplementary figures and images for: Chromatin Loops as Allosteric Modulators of Enhancer-Promoter Interactions
Source: PLoS Comput Biol. 2014 Oct 23;10(10):e1003867. doi: 10.1371/journal.pcbi.1003867 (PMC4207457; doi:10.1371/journal.pcbi.1003867)

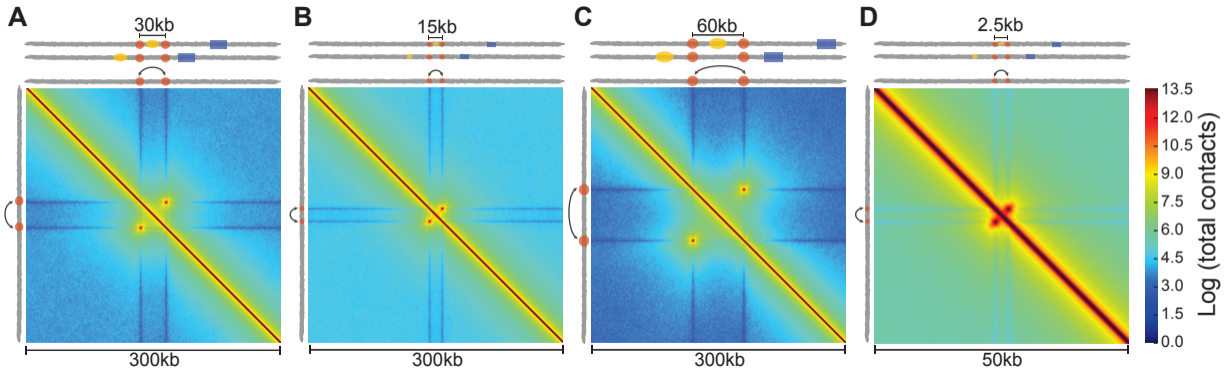

Supplement: Figure S1 — Effects of loop size. Schematics show insulation and facilitation arrangements including the enhancer (yellow), the promoter (blue), and the loop bases (orange) for the heatmaps below. In all cases the main qualitative features remain the same. (A) A 300 kb by 300 kb heatmap for a 30 kb loop, as shown in Figure 2B. (B) A 300 kb by 300 kb heatmap for a smaller loop of length 15 kb. (C) A 300 kb by 300 kb heatmap for a larger loop of length 60 kb. (D) A 50 kb by 50 kb heatmap for a very small loop of length 2.5 kb. In this simulation only, each monomer represents 250 bp rather than 500 bp of a more flexible fiber (k = 2, see Methods), representing a loosely arranged chromatin fiber. This heatmap indicates that insulation and facilitation may still manifest at smaller genomic distances for a more flexible or loosely packed chromatin fiber, as these changes cause small loops to behave similarly to larger loops. Note the color of the map differs due to the smaller dynamic range in total number of interactions for this shorter chromatin fiber, but the same qualitative features are present. (PDF) [file pcbi.1003867.s001.pdf]

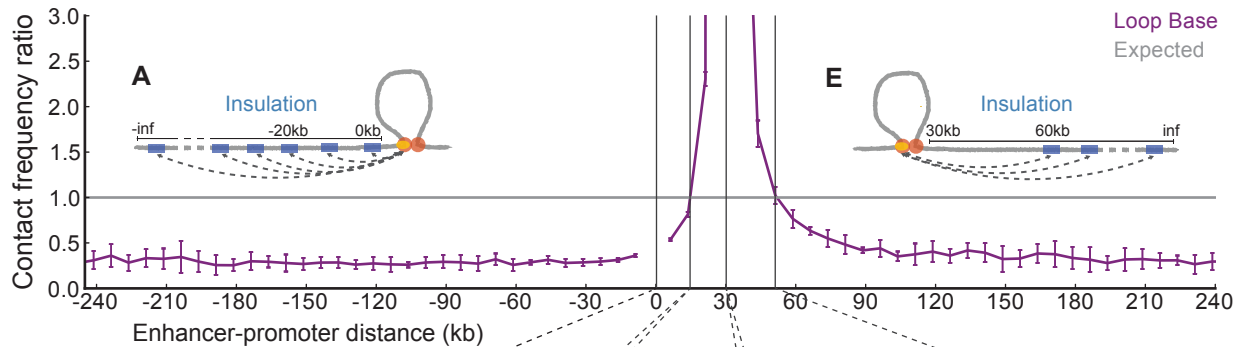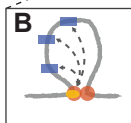

Insulation

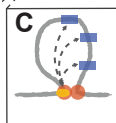

Facilitation

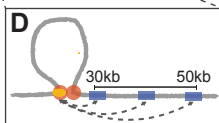

Facilitation

Supplement: Figure S2 — Loop-base profile. Contact frequency ratio of the loop base vs. all other loci (i.e. a 4C-like profile); an enhancer placed at one loop base (0 kb) displays a complex pattern of insulation and facilitation, which we summarize in terms of five regions (A–E). The x-axis shows the upstream or downstream distance to the loop base where this enhancer is placed; note the position of the other loop base is at 30 kb. The y-axis is truncated at contact frequency ratios of 3.0, as when both the enhancer and promoter are positioned at loop bases (i.e. x = 30 kb), the magnitude of facilitation is very large since the loop bases are always in contact. (A) Insulation of the loop base from upstream regions of chromatin. (B) Intra-loop insulation when E-P distance is less than half the loop size. (C) Intra-loop facilitation when E-P distance exceeds half the loop size. (D) Facilitation when the E-P distance slightly exceeds the loop size. (E) Insulation of the loop base from distal downstream regions of chromatin. (PDF) [file pcbi.1003867.s002.pdf]

**A**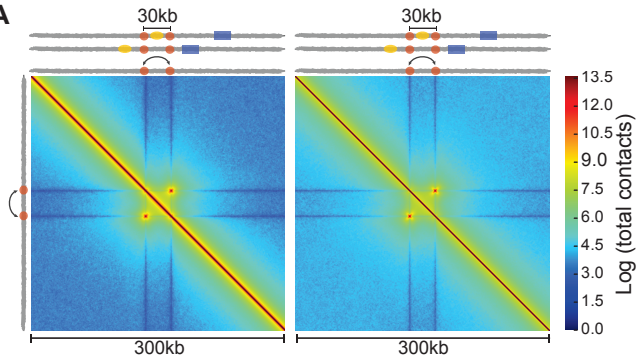**B**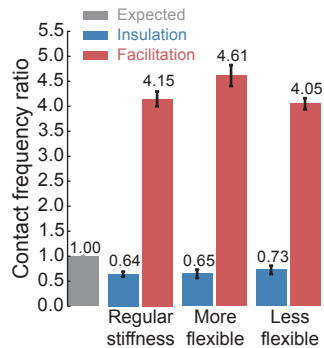**C**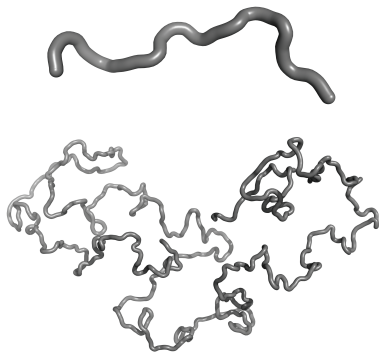**D**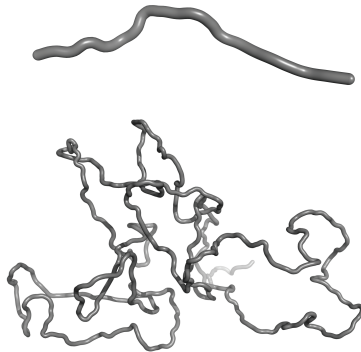

Supplement: Figure S3 — Effects of chromatin fiber flexibility. (A) Heatmap on left displays log (total # of contacts) for simulations with a more flexible polymer and standard parameters: 30 kb chromatin loop, 2% density, fiber crossing (topoisomerase activity). On the right is a heatmap for the less flexible polymer. In both cases, the loop features observed in Figure 2B are still present. (B) Bar plot shows insulation and facilitation: at the stiffness presented in the main figures, for a more flexible polymer, and for a less flexible polymer. (C) (top) shows a 20 monomer or 10 kb stretch from a conformation of a more flexible polymer. (bottom) shows a 500 monomer or 250 kb region from a conformation of a more flexible polymer. (D) Same as (C), but for a less flexible polymer. Note the smoother appearance of the less flexible chromatin fiber conformation. (PDF) [file pcbi.1003867.s003.pdf]

**A**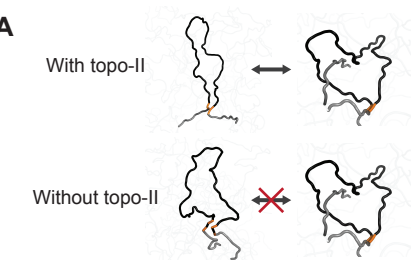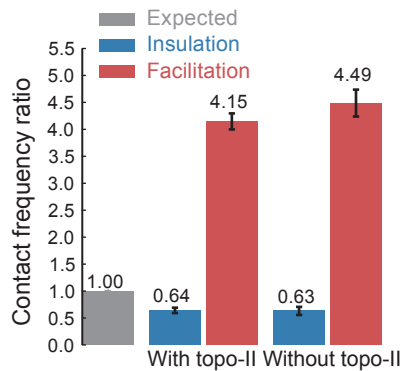**B**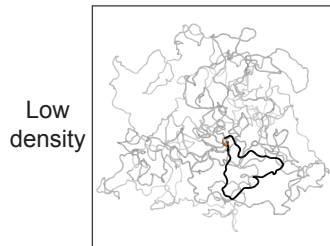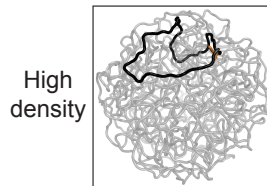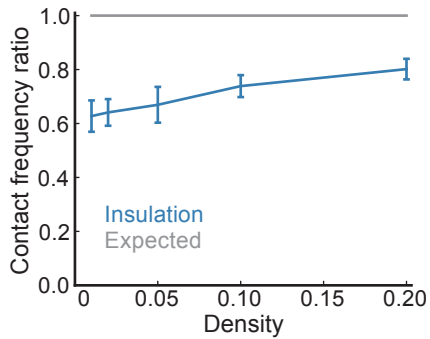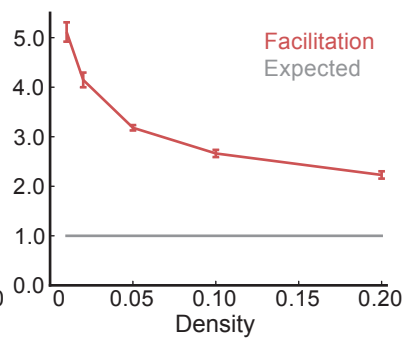

Supplement: Figure S4 — Effect of topoisomerase and chromatin density on local loop-mediated interactions. (A) Effect of topological constraints on insulation and facilitation. With topo-II, there are no topological constraints and a conformation without chromatin threaded through the loop can convert to a conformation with chromatin threaded through the loop. Without topo-II (with topological constraints), chromatin fibers cannot cross and the two conformations cannot interconvert. Bar plot shows the contact frequency ratio for an E-P genomic distance of 50 kb. (B) Effect of density on insulation and facilitation; bar plots show results for 50 kb E-P genomic distance. (PDF) [file pcbi.1003867.s004.pdf]

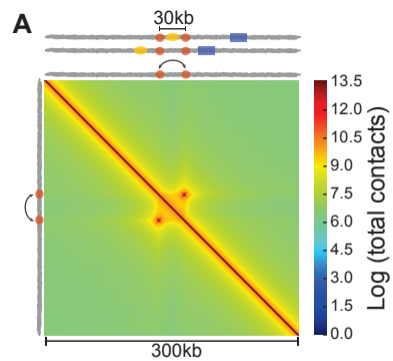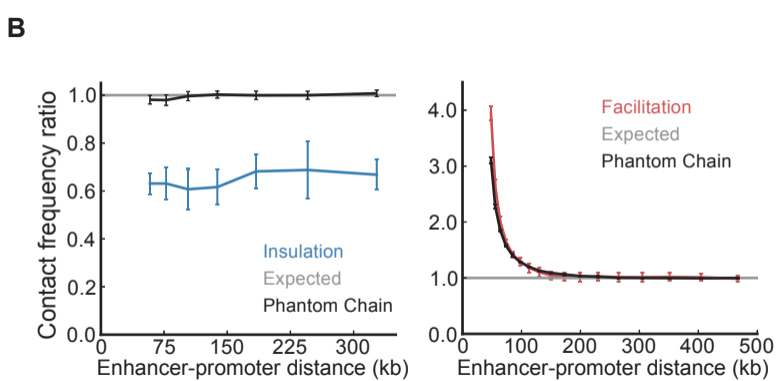

Supplement: Figure S5 — Effects of phantom polymer chain. (A) Heatmap for phantom polymer chain with a 30 kb loop, where insulation and facilitation arrangements are shown as in Figure 2B . The vertical and horizontal stripes of depleted interactions are almost non-existent, indicating dramatically reduced insulation. (B) Bar plot displays insulation and facilitation for the regular scenario (Figure 2B ) on the left and the phantom chain on the right. Facilitation is slightly diminished, whereas insulation completely disappears. (PDF) [file pcbi.1003867.s005.pdf]

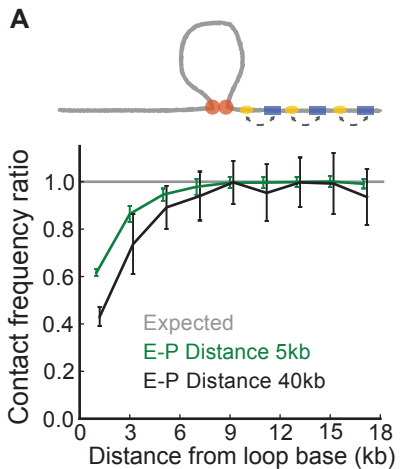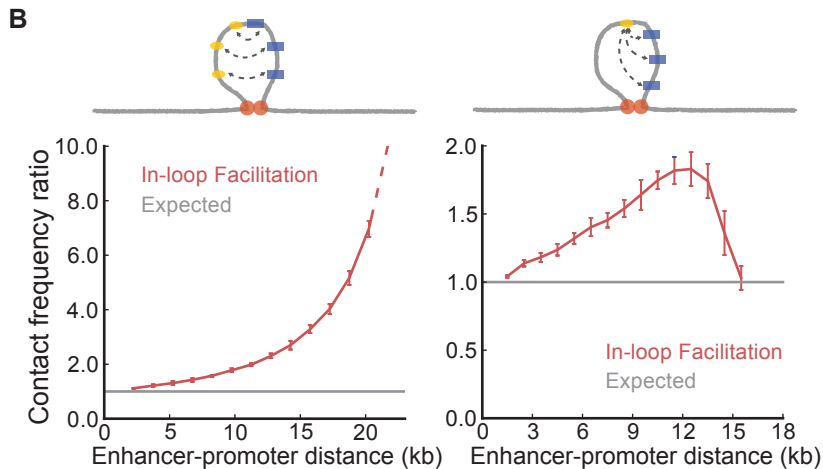

Supplement: Figure S6 — Loop shadowing and intra-loop facilitation (A) Regions immediately outside the loop are also sterically excluded by the loop; in other words the loop's steric “shadow” can cause insulation when the E-P pair is near, but outside, of the loop. Note the black line has been slightly offset, so that error bars are visible. (B) (left) Intra-loop facilitation when the E-P pair is positioned symmetrically within the loop. When E-P distances are much less than the loop size, the loop has a negligible influence on their contact frequency, and the contact frequency ratio is ∼1. However the magnitude of facilitation increases very quickly as E-P distance approaches the loop size because the loop bases are always in contact (corresponding strong peak in Figure S2 at 30 kb = loop size). Note truncated y-axis (at contact frequency ratios of 10.0). (right) Asymmetric E-P placement with increasing E-P distance, where the enhancer stays in the middle of the loop, while the promoter moves towards the loop base. Intra-loop facilitation drops off approaching 15 kb (half of the loop size), due to a superposition with the insulating properties of the loop bases. (PDF) [file pcbi.1003867.s006.pdf]

**A**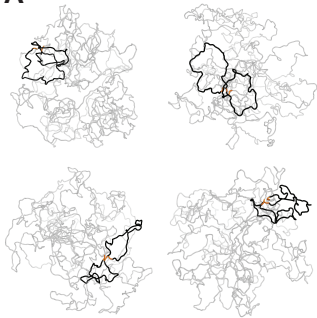**B**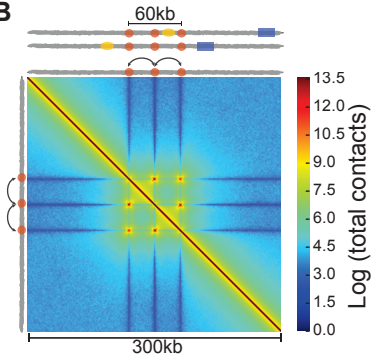**C**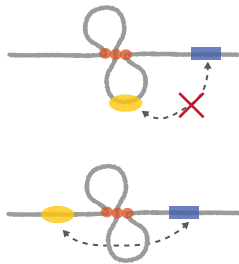

Supplement: Figure S7 — Effects of two consecutive loops. (A) Four sample polymer conformations from simulations of the two-loop system with loops (black) and loop bases (orange) highlighted. (B) Heatmap which shows log (total # of contacts) for the two-loop system. Each loop is 30 kb in the 300 kb by 300 kb region shown. The four red dots closer to the diagonal are the direct interaction of the loop bases from the formation of two loops. The two, weaker, red dots further from the diagonal are the interaction between the base at the start of the first loop and at the end of the second loop. The horizontal and vertical stripes of darker blue are indicative of strong insulation. Annotations show two loops formed from three bases (orange) along with the insulation and facilitation E-P placements. (C) Schematics of E-P arrangement for the two-loop system (top) insulation, indicated the red “X”, (bottom) facilitation. (PDF) [file pcbi.1003867.s007.pdf]
